# Machine learning methods to predict child posttraumatic stress: a proof of concept study

**DOI:** 10.1186/s12888-017-1384-1

**Published:** 2017-07-10

**Authors:** Glenn N. Saxe, Sisi Ma, Jiwen Ren, Constantin Aliferis

**Affiliations:** 1 Department of Child and Adolescent Psychiatry, New York University School of Medicine, One Park Avenue, New York, NY 10016 USA; 2 Institute for Health Informatics and Department of Medicine, University of Minnesota, 330 Diehl Hall, MMC912, 420 Delaware Street S.E, Minneapolis, Minnesota, Mpls, MN 55455 USA; 3 Department of Child and Adolescent Psychiatry and Center for Health Informatics and Bioinformatics, New York University School of Medicine, One Park Avenue, New York, NY 10016 USA; 4 Institute for Health Informatics, Department of Medicine, and Data Science Program, University of Minnesota, Minneapolis, MN USA; 5 Department of Biostatistics, Vanderbilt University, 330 Diehl Hall, MMC912, 420 Delaware Street S.E., Mpls, MN, Nashville, TN 55455 USA

**Keywords:** Traumatic stress, PTSD, Machine learning, Informatics, Child & Adolescent psychiatry

## Abstract

**Background:**

The care of traumatized children would benefit significantly from accurate predictive models for Posttraumatic Stress Disorder (PTSD), using information available around the time of trauma. Machine Learning (ML) computational methods have yielded strong results in recent applications across many diseases and data types, yet they have not been previously applied to childhood PTSD. Since these methods have not been applied to this complex and debilitating disorder, there is a great deal that remains to be learned about their application. The first step is to prove the concept: Can ML methods – as applied in other fields – produce predictive classification models for childhood PTSD? Additionally, we seek to determine if specific variables can be identified – from the aforementioned predictive classification models - with putative causal relations to PTSD.

**Methods:**

ML predictive classification methods – with causal discovery feature selection – were applied to a data set of 163 children hospitalized with an injury and PTSD was determined three months after hospital discharge. At the time of hospitalization, 105 risk factor variables were collected spanning a range of biopsychosocial domains.

**Results:**

Seven percent of subjects had a high level of PTSD symptoms. A predictive classification model was discovered with significant predictive accuracy. A predictive model constructed based on subsets of potentially causally relevant features achieves similar predictivity compared to the best predictive model constructed with all variables. Causal Discovery feature selection methods identified 58 variables of which 10 were identified as most stable.

**Conclusions:**

In this first **proof-of-concept** application of ML methods to predict childhood Posttraumatic Stress we were able to determine both predictive classification models for childhood PTSD and identify several causal variables. This set of techniques has great potential for enhancing the methodological toolkit in the field and future studies should seek to replicate, refine, and extend the results produced in this study.

**Electronic supplementary material:**

The online version of this article (doi:10.1186/s12888-017-1384-1) contains supplementary material, which is available to authorized users.

## Background

More than 20% of children in the United States will experience a traumatic event before they are 16 years old [1, 2]. Of those who experienced a trauma, between 10 and 40% [3–5] will develop Posttraumatic Stress Disorder (PTSD) [6], a disorder that results in significant functional impairment and may have deleterious consequences for brain development [7–9]. The early identification of a child’s level of risk – and specific vulnerabilities – opens the possibility of preventative intervention tailored to the child’s specific needs. Therefore, the ability to predict risk for PTSD from the time of the trauma is extremely important. Unfortunately, the extant research literature has been unsuccessful in reliably identifying a set of risk factors for PTSD common to all traumatized children or specific sets of risk factors that may allow the individualized treatment of a child based on their risk [10, 11]. This limited progress in the field points to the need to identify and apply new methods that might provide improved ways to conduct research towards the reliable and accurate identification of risk factors for childhood PTSD.

This article describes a study of risk for PTSD in acutely traumatized children that – for the first time – employs Machine Learning (ML) computational methods of in order to determine if these methods can identify variable sets and models that predict the development of PTSD. As will be detailed, we believe ML may offer much needed advantages for advancing the field of risk factor research for childhood PTSD because of its track record for these purposes in other fields [12–26] and its success in its first application to adult PTSD in the pages of this journal [27].

### The current state-of-the-science for child PTSD risk factor research

Over the last twenty years, a sizeable literature on childhood PTSD risk factors has accumulated. Unfortunately, the literature has not converged on a set of risk factors that accurately identify risk or inform care. Meta-analytic studies have concluded that many of these risk and protective factors have small effect sizes for traumatic stress, and the results on these effect sizes are not consistent, between studies [10, 11]. Trickey and colleagues published the most definitive meta-analysis to date, examining 64 studies of risk factors for traumatic stress in 32,238 children (aged 6–18 years) over a 20-year period (1990–2009). Of note, only 25 risk factors were examined, as these were the only ones reported in more than one study and only six risk factors were assessed in more than 10 studies. Ten risk factor variables showed medium to large effect sizes, but four of these were only examined in two studies and three were found to have inconsistent effect sizes across studies. Only one risk factor was found to have a large effect size in a large number of studies (post-trauma psychological problem) [10].

### The fit between the complexity of childhood PTSD and the data analytic methods used to determine risk

A key observation relevant to our study is that from a mathematical perspective, a risk factor is a variable that conveys statistical information about the likelihood of the phenotypic response of interest. The discovery of accurate risk factors from data critically depends on the choice of data analytic approach. A large literature on feature selection methods developed and applied in various fields over the last several decades shows that different features (i.e., risk factors in our study) and models using those features will be selected by different data analysis methods. For a broad introduction to modern feature selection see Guyon and Elisseeff [28]. Tsamardinos and Aliferis showed that there cannot be a uniformly “best” feature selection method, and that feature selection methods must be designed for specific requirements [29]. For example if a maximally compact risk factor set is desired among the sets that are maximally predictive, a feature selector that discovers Markov Boundaries has to be employed (more on this later in the manuscript). This property of Markov Boundaries is intrinsic to the system under study and does not change by the method a researcher chooses to study the system.

In the majority of traditional data analysis in psychiatry and in PTSD research, including all of the 64 studies described in the Trickey et al., risk factors are discovered using either univariate association, or stepwise procedures within various forms of the General Linear Model (GLM) family of multivariate analysis methods [30–32]. The GLM refers to a broad category of established statistical models based on regression that includes Analysis of Variance, Linear Regression, Logistic Regression, and Poisson Regression among other types of classical multivariate analysis. These approaches, however, do not guarantee predictive optimality and do not guarantee parsimony in a data analysis-independent manner. Very importantly, the results are tied to the specific method used for analysis and are essentially an artifact of the analysis method used and not an intrinsic property of the system under study. In addition, robust understandings of childhood PTSD, in all likelihood, involve the influence of a great many variables from a diversity of modalities (e.g. genomic, neurologic, physiologic, social, developmental) and, most importantly, the interaction between these variables. Traditional data analytic methods (e.g., classical regression and sister methods from the GLM family, but also clustering and decision tree methods) impose considerable restrictions on the number of variables that can be used in a given analysis and, especially, the analysis of interactions. Another, major, problem with older methods concerns their limited ability to shed light on causality when the data does not come from randomized experimental designs. Experimental designs however are unethical in (non-animal) risk factor research related to trauma (i.e. assigning a child to a trauma exposure condition). It is for this reason that all human risk factor research for childhood PTSD is correlational. The essential correlational nature of this research has considerable implications for prevention. An identified risk factor can represent a promising target of preventative intervention if – and only if – it represents a cause of the phenomenon it is thought to influence. ML computational techniques offer advantages for each of the above limitations of older/classical data analytic methods [33–36]. For example, the aforementioned Markov Boundary feature selection methods will also find the local causal neighborhood of the response variable in most distributions [29, 37].

Newer advances in data analysis contributed by the field of Machine Learning greatly extend the researchers’ ability to make meaningful discoveries also by:

1. Enabling accurate and reliable prediction using data with very large numbers of variables and small sample sizes.
2. Avoiding the significant hurdles of estimating accurate variable coefficients and of modeling the data generating processes by directly building accurate predictive classification models for phenomena of interest and testing the reliability and accuracy of these models without the need for data-generative models and accurate coefficient estimation.
3. Enabling causal inference within non-experimental data sets.

The conceptual foundations for these algorithms are based on thoroughly validated body of work exemplified by the Nobel and Turing award winning work of Herbert Simon, of Turing award winner Judea Pearl, and of Nobel laureate Clive Granger, among other pioneers of non-experimental causal discovery [38–42]. On a purely empirical and practical level, research using ML methods has met exceptional success in a wide range of scientific and technological fields [34], and it is beginning to penetrate the domain of clinical science, including the fields of psychiatry and pediatrics. ML has demonstrated utility in a variety of applications including the accurate classification in pediatric disorders such as epilepsy, asthma, heart disease, and head injury [20–23]. Within psychiatry, ML has been successfully used in the predictive classification of autism, attention deficit hyperactivity disorder, and schizophrenia [24–26]. ML has recently been used to predict PTSD in acutely traumatized adults [27, 43]. It has not yet been used to predict PTSD in children or to identify causal processes for PTSD, however. The possibility of using ML to identify causal processes – initiated shortly after exposure to trauma – has important implications for prevention. The detection of such causal processes may thus identify promising targets for preventative intervention.

The current study addresses two broad hypotheses:

*Hypothesis 1*: ML methods can identify an accurate and reliable predictive classification model for childhood PTSD, from variables measured around the time of trauma.

*Hypothesis 2*: ML methods can identify variables that not only have predictive value for childhood PTSD, but can also identify those with causal influence.

## Methods

### Data set

The research data set comprises information on 163 children aged 7–18 collected as part of a National Institute of Mental Health funded study (R01 MH063247) on risk factors for PTSD in children hospitalized with injuries. This study was funded in 2002 to gather information – using the best methods available at that time – about risk for PTSD from children hospitalized with injuries. Thus variables originally selected for this study (and now contained in this data set) include a broad range of risk variables hypothesized (at the time) to predict the development of PTSD. The basic study design is as follows: injured children were assessed within hours or days after their hospitalization and reassessed three months following discharge. The data set includes 105 variables measured during the hospitalization that will be considered as possible features for our predictive model. These ‘feature’ variables belong to such domains as childhood development, demographics, parent symptoms, stress, magnitude of injury, candidate genes, neuroendocrine and psychophysiologic response, and child symptoms and functioning. The target variable is a UCLA PTSD Reaction Index Score of 38 or greater, measured three months after hospital discharge. This cutoff score is based on a high level of symptoms of PTSD and is strongly related to a DSM IV diagnosis of PTSD [44]. Due to space restrictions, we provide detailed information about each of the variables in Additional file 1. The data set can be found in Additional file 2.

### ML methods for predictive classification of childhood PTSD

**Fig. 1:**
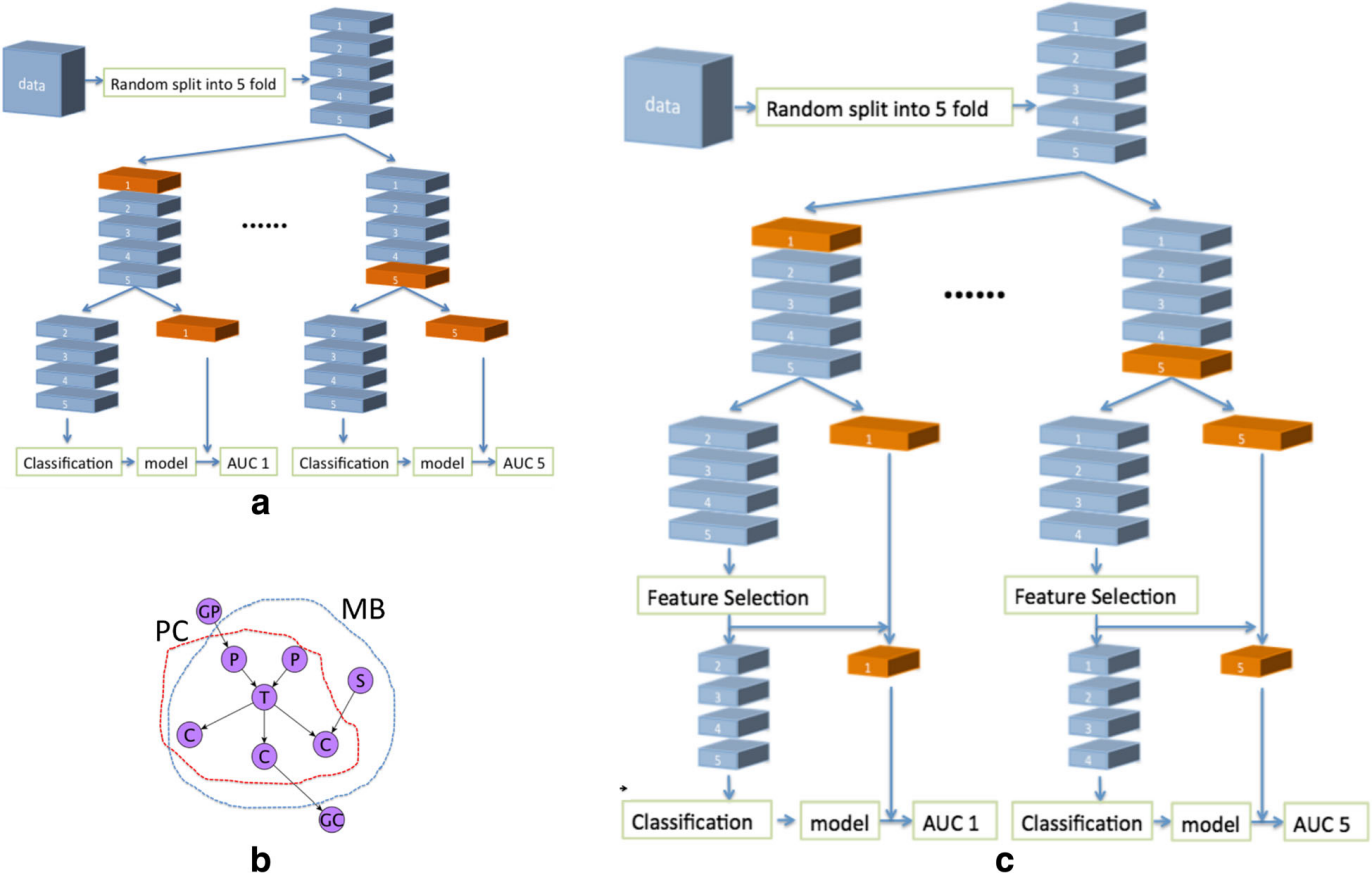
Flowchart for cross validation. **a** The 5-fold cross validation process. The widths of the *rectangular data boxes* represent the dimension of features. The heights of the rectangular data boxes represent the dimension of subjects. An *orange rectangular* data box represents a testing data set. **b** A simple example of Causal Network. Node T represents the “target” (i.e. response variable); Nodes P represent parents of target; Nodes C represent children of the target; Node S represents spouse of target. MB is the Markov Blanket comprising parents, children and spouse; PC is the parents and children set. For details please see text (**c**) 5-fold cross validation process with Feature Selection

To test Hypothesis 1, we apply ML predictive classification methods, as pictorially summarized in Fig. 1. The analysis protocol simultaneously performs **Model Selection and Error Estimation**. Model Selection refers to examining which parameters of a given classifier are best for the data at hand. Error estimation is the calculation of the expected predictivity of the best model identified when this model will be applied in data from the same population of subjects. The protocol is called Repeated Nested N-Fold Cross Validation (RNNCV), and it has become a standard way to apply complex ML analysis in a number of fields because it has a number of important properties: (a) it provides unbiased estimates of generalizability of the best model found; (b) it reduces variance of these estimates; (c) it is fully automated which allows for powerful model discovery and also for testing the analysis for overfitting and statistical significance. The RNNCV is described in detail in [45].

Very briefly, RNNCV divides data into two components: i. a training data set (comprising 80% of subjects in our case) and ii. a testing data set (comprising the remaining 20% of subjects). This split is performed N times (*N* = 5 in our study) so that all data is used exactly once as test data. The process is repeated multiple times (30 times in our case) to reduce data split variance. This is the “outer loop” that performs the Error Estimation. Within each training data set a second round of nested cross validation is performed (each training set divided in N-1 train-train and train-test subsets). Different algorithms and parameters are used to build models with train-train data and test them on train-test data. This inner loop is performing the Model Selection component of the analysis. By comparing results across data splits, the protocol can find the most powerful parameters for individual classifiers. Then, these are used to build models in the outer loop and evaluate future predictivity. Because – at each application of a model on a test set – the model had not been previously ‘exposed’ to the test data, the error estimates are unbiased. We measure the performance of the classification model by evaluating the *Area Under the Receiver Operating characteristic Curve (AUC)* [46] which measures classification predictivity over the full range of cutoffs (each cutoff representing a different tradeoff between sensitivity and specificity).

#### Choice of classifier families

Three state-of-the-art ML classifiers (and variants) are used: Support Vector Machines (SVMs), Random Forests, and Lasso (Regularized Regression). The classification models separate subjects into two groups: 1. Those whose UCLA PTSD RI score is greater than or equal to 38 (PTSD Group), 2. Those whose UCLA PTSD RI score is less than 38 (Non-PTSD Group).

SVMs identify a geometrical hyperplane that maximizes the margin between these two subject groups. SVM classification has several appealing properties: First, it has no restrictive assumptions about the distribution of data. Second, subjects that form the decision boundary for classification – the “support vectors” – are the free parameters in an SVM model independent of the total number of variables. Statistical leaning theory of SVMs shows that the error of SVMs depends on the number of support vectors making this classifier extremely robust to modeling when the number of dimensions is very large and the number of subjects is very small [47]. SVMs also have powerful ways to build non-linear discriminatory functions examining huge numbers of interaction effects in seconds and without overfitting, by doing this implicitly. In our study, we applied both linear and non-linear (RBF and Polynomial Kernel) SVMs.

The second type of classifier is Random Forests [48]. Random Forest classification ensembles multiple decision trees, performs internal feature selection, and error estimation, and assigns a class to new cases based on the mode over the distribution of predictions predicted by the individual trees in the forest.

The third type of classifier is Lasso. Lasso is a combination of classical Regression with Regularization. Regularization (also employed by SVMs) refers to imposing a restriction on the weights of all variables so that the sum of squared weights is minimized while the model fit to the data is simultaneously maximized. Both Lasso and SVMs tend to implicitly eliminate unnecessary variables by settings their weights to zero.

Thus, we examine predictive performance using five approaches to ML predictive classification: 1. SVM Linear, 2. SVM RBF, 3. SVM Polynomial Kernel, 4. Random Forest, and 5. Lasso.

We also examine the predictive performance using more conventional classification methods in order to determine the ‘added value’ of the newer ML methods for predictive classification. Accordingly, in addition to ML models we also build predictive models for the PTSD target using: 1. Logistic Regression alone, and 2. Logistic Regression coupled with Stepwise (forward-backward) Feature Selection.

Finally, we examine the predictive performance of all seven classifiers (five ML and two conventional), when each classifier is coupled with ML feature selection.

#### Classification performance metric

As described, AUC is used to evaluate the prediction performance of the classification model. It is based on the Receiver Operating characteristic Curve (ROC): a plot of the sensitivity versus 1-specificity by varying the classification cut off point between one group and another group [46]. AUC scores range between 0 and 1, with higher scores corresponding to better classification performance. An AUC score of 0.5 corresponds to random assignment of subjects to classification groups (from 0.5 to 1, predictivity increases for classifying to the correct class; from 0.5 down to 0, predictivity increases for classifying to the wrong class, i.e., 1 = perfect classification to the correct class, 0 = perfect classification to the wrong class; 0.5 = random classification normalizing for classes difference in sample).

### Testing for overfitting and *p*-values for overall predictive signal: Label shuffling test

Label Shuffling permutation testing is used to (a) Test the null hypothesis that there is no signal in the data (i.e., equivalently determine whether the model will achieve more accurate prediction than what would be expected by chance if the data does not have any predictive information about PTSD) and (b) Quantify the degree of overfitting when sampling under the null hypothesis. If the mean AUC under the null hypothesis is not 0.5, then the analysis protocol is biased by an amount equal to the distance from 0.5.

The label reshuffling procedure is implemented by randomly shuffling the PTSD outcomes within the data set and then conducting the same ML predictive analytic procedure on this ‘null’ data set, as described previously. This Label Shuffling process is repeated by permutation 400 times to create 400 data sets sampled under the null hypothesis for the ML predictive classification analysis. The mean AUC for the 400 random data sets is derived and the distribution of AUCs of the shuffled data sets is compared to the AUC of the actual data set to calculate the probability (one-sided *p*-value) of deriving an equal or higher AUC score than that observed from the original data (without Label Shuffling).

### ML methods to select variables with causal relations to PTSD

Thus far, the ML analysis seeks to build a predictive classification model for PTSD from all the features in the data. There are reasons to try to *select* a set of *features*, amongst all those measured, and to determine the reliability and accuracy of this smaller set of predictive features compared to the entire model. The main rationale for *Feature Selection* in this study is (a) to identify highly predictive and compact feature sets (Hypothesis 1) and (b) to identify a set of features with possibly mechanistic (causal) influence on PTSD (Hypothesis 2). Several high quality causal discovery Feature Selection methods are available. The one we employ is called semi-interleaved HITON-PC without symmetry correction [41, 42], and we use this method within the ML predictive classification approach to identify possible causal features for PTSD that also form an accurate predictive classification model, as explained before.

ML methods used to test Hypothesis 2 are the same as those used to test Hypothesis 1, except that a classification model is built with variables selected by the HITON-PC Feature Selection method rather than from all features in the data set. HITON-PC performs causal discovery through learning local causal networks and approximating Markov Boundaries [41, 42]. A Causal Network is a graph that represents the causal relations between a set of random variables. In this study, a causal network would represent the causal relations from sets of features in the data set and our response variable (the “target”) – whether a child would have a UCLA PTSD RI score of 38 or greater. An example of a simple causal network is shown in Fig. 1b. Each node represents a random variable. An edge between two nodes means that there is a direct causal relationship between them. In our study, the nodes are either features measured in the hospital or the target measured three months later. In Fig. 1b the node labeled with T represents the target (i.e., the response variable, PTSD in our study); the nodes labeled with P represent direct causes (in causal graph terminology, called the “parents”) of the target; C represent direct effects (called the “children”) of the target; and S represent the direct causes of the direct effects (called the “spouses”) of the target. Under broad distributional conditions, the Markov Boundary of the target is the set of variables comprising the parents, children and spouses of the target. Variables in the Markov Boundary have a fundamental property: they are the minimal set of variables that capture all the available non-redundant predictive information in the data regarding the target that we want to predict [41, 42]. Accordingly, we aim to identify variables/features that are in the Markov Boundary of the target and only use those variables to predict the target. It is important to note that HITON-PC does not find the full Markov Boundary, but only the direct causes and direct effects of the response variable. Since the response variable in this study (PTSD) is a terminal one, there are no spouses. Therefore, it is not necessary to use algorithms that will discover the full Markov Boundary. In this study, because PTSD is terminal and thus has no children, HITON-PC can be expected to contain only the direct causes of PTSD and that set to approximate the full MB properties.

### Stability assessment

HITON-PC is asymptotically correct, but in modest samples sizes such as in the present study, further filtering may be helpful for identifying highly robust causes of the target. Specifically, we filter the results from HITON-PC by bootstrapping stability analysis as follows: 100 bootstrap samples of the data set are created and HITON-PC feature selection is applied to each. The frequency of each feature/variable selected by HITON-PC out of the 100 bootstrap samples is recorded, and we focus on causal variables with the highest stability across these bootstrapped samples. Friedman and colleagues detail the rationale and application of stability filtering for these purposes [49].

### Missing data

The percentage of missing data for each variable was calculated and variables missing greater than 50% of subject data were eliminated. We then use the Knnimpute method [50] to impute missing data in an unsupervised manner. Knnimpute is a non-parametric nearest neighbor method to impute data. Using this method, variables are also normalized to a range of 0 to 1 to reduce data artifacts due to differences in scaling.

## Results

Eleven of the 163 (7%) of injured children received a score of 38 or higher on the UCLA PTSD Reaction Index and would be classified within our PTSD Group.

### Performance of predictive classification models for childhood PTSD

**Fig. 2:**
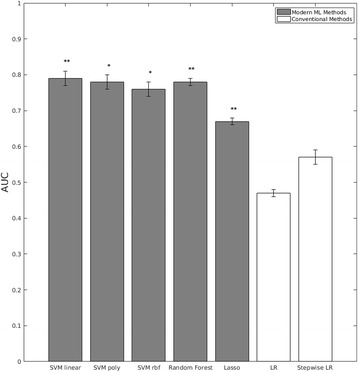
Performance of Predictive Classification Methods constructed with all variables. Predictive performance that are significant at *p* < 0.05 is labeled with *, predictive performance that are significant at *p* < 0.01 is labeled with **. The *dotted line* placed at AUC = 0.5 indicates the expected performance under the null hypothesis (no signal in the data)

Hypothesis 1 is tested by examining the mean AUC results for the classification of PTSD using each of the five ML classification methods (SVM linear, SVM poly, SVM RBF, Random Forest, Lasso), compared to the two conventional methods (Logistic Regression, Stepwise Logistic Regression). Figure 2 shows the results of this analysis. As can be seen, using each of the five ML classification methods, a classification model was obtained with considerable predictive signal (best model: mean AUC = 0.79). These results are much stronger than the performance yielded by the conventional classification methods where predictive signal was at the chance level. Randomly shuffling labels diminished performance significantly for the five ML classification methods (Table 1).

**Table 1:** Performance of classifiers and feature selection methods

Classifier		All features	Feature selection with HITON-PC
SVM linear	observed data	0.79 (0.02)**	0.68 (0.04)*
	label shuffling	0.50 [0.32 0.71]	0.50 [0.36 0.67]
SVM poly	observed data	0.78 (0.02)*	0.68 (0.04)
	label shuffling	0.50 [0.31 0.71]	0.50 [0.34 0.71]
SVM RB	observed data	0.76 (0.02)*	0.68 (0.04)
	label shuffling	0.50 [0.36 0.70]	0.50 [0.35 0.69]
Random forest	observed data	0.78 (0.01)**	0.74 (0.01)*
	label shuffling	0.50 [0.33 0.67]	0.50 [0.33 0.73]
Lasso	observed data	0.67 (0.01)**	0.74 (0.01)*
	label shuffling	0.50 [0.44 0.57]	0.50 [0.35 0.68]
Logistic Regression (LR)	observed data	0.47 (0.01)	0.72 (0.01)
	label shuffling	0.50 [0.35 0.64]	0.51 [0.32 0.74]
Stepwise LR	observed data	0.57 (0.02)	0.72 (0.02)*
	label shuffling	0.51 [0.39 0.64]	0.49 [0.31 0.71]

The performance (measured as Area Under the ROC Curve) of individual classifiers and feature selection methods in the observed data and under the null hypothesis of no signal in the data (estimated with label shuffling). For observed results the mean and (standard deviation) were presented. For the label shuffling, mean and [95% confidence interval] were presented. Predictive performance that are significant at *p* < 0.05 is labeled with *, predictive performance that are significant at *p* < 0.01 is labeled with **

### Performance of predictive classification models – With causal discovery feature selection - for childhood PTSD

**Fig. 3:**
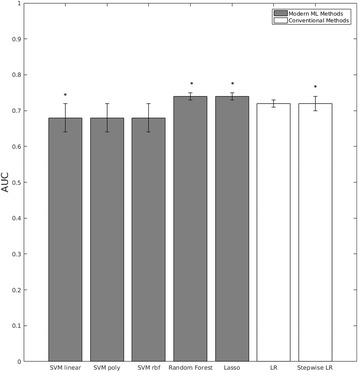
Performance of Predictive Classification Methods with HITON-PC Causal Discovery Feature Selection. Predictive performances that are significant at *p* < 0.05 are labeled with * and predictive performances that are significant at *p* < 0.01 are labeled with **. The dotted line placed at AUC = 0.5 indicates the expected performance under the null hypothesis (no signal in the data)

Examining the mean AUC results by applying the HITON-PC causal discovery feature selection method addresses directly Hypothesis 1 and indirectly Hypothesis 2. Figure 3 shows the results of these analyses using each of the five ML classification methods (SVM linear, SVM poly, SVM RBF, Random Forest, Lasso) and the two conventional methods (Logistic Regression, Stepwise Logistic Regression). In this case, strong predictive performance was yielded in models obtained by all seven methods (including the two conventional methods). When the labels were randomly shuffled, the predictive performance was entirely diminished for all seven classification methods (Table 1).

Figure 3 shows the distribution of predictivity estimates across the RNNCV (nested 5-fold cross validation AUC estimate over 30 repeated runs of cross validation). As can be seen from this distribution, all yielded AUC estimates are greater than chance with a mean AUC of 0.79.

**Fig. 4:**
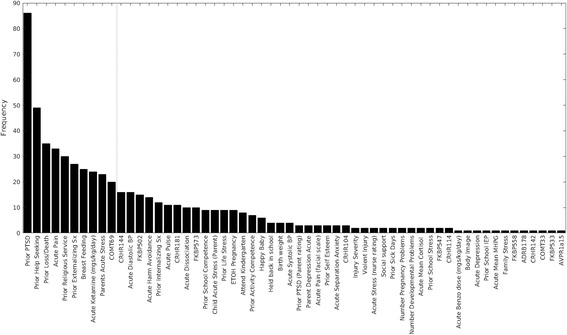
Frequencies of causal variables selected out of 100 bootstrap samples. (Variables of frequency greater than 20% to the left of *dotted line*)

### Identifying the most stable causal features for childhood PTSD

As described previously, the utility of ML causal discovery feature selection methods will be based on examining the variables selected in resulting causal predictive models for their potential to identify possible preventative intervention strategies (since they are expected to represent mechanisms that influence PTSD). Fifty-eight variables were discovered to contribute to the performance of these causal models. We then sought to identify the most stable of these causal variables in order to identify those might be reliable targets of preventative intervention. These were identified in the bootstrapping analysis. The results of the bootstrapping stability assessment are shown in Fig. 4 with the frequency of selection for each of the 58 causal variables within the 100 bootstrapped samples of our data set. The variables selected by HITON-PC were ranked by the frequency that a given variable appeared in one or more of the 100 bootstrapped samples. This frequency ranged from 1 to 88. Ten causal variables appeared in more than 20 of the bootstrapped samples and are operationally considered the most stable causal variables for our purposes. Figure 4 identifies these 10 variables, and each will be reviewed in the Discussion section. Details of how these variables were measured are offered in Additional file 1. We note that all features selected by HITON-PC must pass several univariate and multivariate tests for statistical significance in order to be output and are furthermore corrected for false positives due to multiple comparison testing by the algorithm’s inherent control for such false positives. Thus, low stability levels are not indicative of the variables being uninformative or random but are mostly due to information overlap between variables. See Additional file 3 for further technical details on the interpretations of HITON-PC output, causal validity of the identified variables, and SVM model weights.

## Discussion

In this first study of the application of ML methods for the prediction of childhood PTSD, several significantly predictive classification models were discovered from variables measured around the time of trauma. The AUC performance results support this conclusion. The performance results of the SVM models using causal feature selection also yielded strong predictive performance, which combined with the terminal nature of the PTSD variable and the theoretical correctness of HITON-PC for discovering the causal causes and effects of the response variable, as well as the Markov Boundary of the response in the absence of “spouse” variables suggest that the identified features are the direct causes of PTSD in this dataset. Label Shuffling Tests showed that the analysis and error estimation protocol is unbiased and that the strongly predictive models had statistically significant predictivities. Thus, this study demonstrated the potential utility of ML methods for childhood PTSD, even with a sample of very modest size. We also successfully applied several safeguards against, and detection procedures for, model overfitting (e.g. using regularized classifiers, applying consistent feature selection procedures, five-fold cross validation, bootstrapping for stability assessment, and label shuffling). Obviously, such results should be replicated and methods refined, but the results should generate confidence in the promise of these methods.

It is also instructive to compare the performance of classical analysis approaches to modern ML ones. Traditional analysis (Logistic Regression) without feature selection was incapable of discovering any predictive signal. Traditional analysis with conventional feature selection (via forward-backward selection) was also incapable of discovering any statistically significant predictive signal (and nominal signal was very weak). When combining traditional LR with modern ML feature selection, robust signal is detected. Similarly replacing traditional regression analysis with regression that is fitted via modern ML regularization (Lasso) robust signal is also detected. These analyses, consistent with the theory of modern statistical machine learning, vividly demonstrate that traditional analysis methods, used for years in the field, cannot cope with high-dimensional large datasets commonly found in present-day research, and that modern methods can provide valuable enhancements.

The performance of the ML predictive models is strongly encouraging. Using causal discovery feature selection, predictive models with strong performance were also identified. Moreover, the specific variables that were found to be most stable in the predictive classification model are interesting and may shed light on the process by which PTSD emerges – and the possibility of prevention – in acutely injured children. In this discussion, we focus on the 10 most stable causal variables. Of note, several (but not all) of these variables have been associated with PTSD in previous correlational studies (e.g. prior PTSD, pain, parent’s acute symptoms, externalizing symptoms, candidate genes) [51–56]. None of the previously described variables, however, have been associated with childhood PTSD in a research design that enables causal inference within a sound ML causal discovery model. We review these causal variables, next.

### Prior PTSD

The causal influence of a child’s history of PTSD may relate to the disordered threat-response system that many describe as underlying traumatic stress psychopathology [57, 58]. In children with disorders of these systems, a new trauma may evoke extreme survival laden emotional states and defensive behavior through threat response systems already potentiated by prior PTSD. These results indicate the importance of assessing prior PTSD in an acutely traumatized child.

### Prior externalizing symptoms

Children who displayed externalizing symptoms prior to the injury were at higher risk for PTSD. Although the association between externalizing symptoms and child PTSD has been reported previously [59], the appearance of this variable in the causal model indicates the importance of assessing prior externalizing symptoms for risk assessment and consideration of the impact of externalizing symptoms on an injured child’s functioning for possible preventative intervention.

### Prior loss

The experience of injury may arouse feelings of grief in the wake of trauma. Injured children may also long for lost loved ones who they depended on in times of stress such as hospitalization after injury. Prior loss has been associated with PTSD in children in correlational studies [60]. This finding indicates the importance of assessing a history of loss in an acutely traumatized child and addressing the child’s grief even when the loss occurred long ago.

### Acute pain

Ongoing pain represents the continuity of the traumatic experience and can evoke continuing memories of the injury, lead to ongoing fear about whether recovery will occur, and may evoke worries about the effectiveness of caregivers (medical and non-medical) for helping with pain and distress. Thus, acute pain can be a marker of risk and a target for preventative intervention.

### Acute stress symptoms in the parent

Parents with acute stress may be less able to attend to a child’s emotional and physical needs. The child’s physical appearance and level of their distress may evoke traumatic stress symptoms in the parent causing them to avoid the child or to become overwhelmed while providing care [52]. The causal relation between parent’s acute stress and the child’s PTSD may indicate the promise of treating the parent, for the prevention of the child’s PTSD.

### Protective factors

A child’s history of *breast-feeding* and *attendance of religious services* were included in the causal predictive model. There is a large literature on the benefits of breast-feeding for promoting a young child’s nutritional status, regulation capacities, and attachment relation [61, 62] but to our knowledge breast-feeding has never before been demonstrated to be protective of PTSD. This finding may contribute to knowledge about the public health importance of breast-feeding. Similarly, there is an important literature about religion and spirituality for fostering a sense of community, social support, and meaning and purpose in life [63, 64] but it, too, has never been documented as protective of PTSD. This finding may suggest a promising approach for prevention for children and families who are inclined towards spirituality and religion.

### Candidate genes

Single Nucleotide Polymorphisms (SNPs) for several genes were included in our causal predictive model (e.g. COMT, CRHR1, FKBP5). These genes have been associated with PTSD in previous research by our team and others [53–56]. These findings indicate that these genes can be used to help assess risk profiles for acutely traumatized children and—because of their causal relations to PTSD—may reveal new possibilities for prevention related to the biological pathways in which these genes form a part.

### Prior help seeking

One variable selected in our model was related to intervention but, paradoxically, it has a *positive* relationship with PTSD. The variable was based on the response to a single question asked about the child on the Diagnostic Interview for Children and Adolescents (DICA) [65]: “Has the child talked to someone (counselor, doctor, rabbi, priest, etc.) about his or her troubles?” Importantly, this question does not distinguish whether the child talked to a mental health professional, a non-mental health professional, or a non-professional about their ‘troubles’ (however defined). Nor does it ask whether the child’s experience of talking about ‘troubles’ lead to any help or relief. Prior negative or neutral experiences may have reduced the likelihood of seeking appropriate help after trauma. Such an interpretation is speculative and more research should be conducted on this issue.

### Ketamine

Ketamine, a glutaminergic agent administered to some children as part of their anesthesiologic protocol was positively related to the development of PTSD. This is a surprising result. Ketamine has recently received attention via its capacity to help treatment-resistant depression [66]. Obviously, such a result may correctly indicate that Ketamine intervention can cause PTSD. This result also reveals a limitation of the causal discovery algorithms we employed. Of the set of measured variables, these algorithms have a strong track record for accurately detecting causes and eliminating the majority of distal causes and all distal confounders. They will, however, allow for some false positives when unmeasured causes exist in the local causal neighborhood of the response variable. It is possible that Ketamine is a proxy for another unmeasured pharmacologic or extrapharmacologic variable that was a true cause of its influence. As we discuss below, techniques that detect local confouders exist, but typically require larger sample size than the sample available for our study (and are also computationally harder to apply to large dimensionalities).

### Clinical implications

In this first application of ML methods to predict risk for child PTSD, we have identified reliable and accurate predictive models and – when applying causal discovery feature selection – we identified risk factors that had face value as causally influencing the development of PTSD. Thus, such findings have important implications for identifying effective preventative interventions and have potential to translate to clinical care. The variables that are contained in the predictive causal model provide all the non-redundant information that that can provide accurate prediction with the accuracy metrics employed (AUC ROC). Thus, our models provide information about the most important variables to assess (and on variables that do not contribute to accurate risk assessment), thus enabling a more efficient clinical assessment process.

The variables identified in the resulting causal predictive model can practically be gathered in clinical settings. Regarding the time needed to gather the information within the risk models: Of the 10 most stable causal features shown in Fig. 4: One feature is a SNP for a gene (COMT69) collected from a saliva sample, one feature is a variable gathered from the child’s medical record (Ketamine dose), and the remaining eight features are gathered from questionnaires or child interviews. We estimate that this information can be gathered in no more than 15 min of a clinical encounter and the benefit yielded may be considerable: accurate knowledge of a child’s risk. Of the remaining forty-two variables in the causal model, twenty are SNPs for seven genes or other biological variables (e.g. cortisol) collected through saliva sample and the rest are either items or scales from questionnaires or information gathered through medical record review. These can also be easily gathered in a short period of time. Although it is more convenient to gather information from questionnaires or interviews than from biological samples, saliva sampling is non-invasive, well tolerated by children and families, and – if biological assessment is focused on the small number of genes/SNPs found to be predictive – the resulting cost is quite inexpensive.

These findings also have implications for the thoughtful allocation of scarce clinical resources that have become a ubiquitous problem in most clinical settings. In such settings, the allocation of clinical resources for care to those most in need (i.e. high risk individuals) is extremely important. If our results hold up to replication, these models can help clinical organizations to identify the children most in need of care and to avoid exposing children who are not at risk to unnecessary care (and potential iatrogenic consequences of such care).

### Limitations

As described, our data support the promise of ML for risk factor research for childhood PTSD, but we note several limitations that future research can address. These include:

- A relatively small sample size was available. Future research with larger samples will be helpful for not only verifying the overall predictivity of our risk factor panel and the generalizability of individual risk factors but potentially produce risk factors and models with even better predictivity. Increased sample size can also allow application of causal discovery techniques that can detect confounding in the data due to unmeasured variables.
- A high PTSD cutoff score was used. A UCLA PTSD RI score of 38 is indicative of a very high level of symptoms. Future studies should evaluate ML in children with more moderate levels of symptoms.
- The sample only included injured children. It will be very interesting for future studies to test generalizability across trauma types.
- PTSD was predicted at three months following trauma. Future research can seek to predict PTSD over a longer duration of time.

### Statistical methods or machine learning?

We wish to emphasize that the present work is not advocating abandoning classical statistical or other data analytic methodologies, but rather to embrace both older and newer method and choose the right methods for the data analytic goals of specific studies. Experts in the field of data analysis appreciate that in certain circumstances, techniques from different fields exhibit similar behavior, reduce to the same discriminant functions, or turn out to give similar results in certain datasets and tasks. A single layer perceptron for example is not fundamentally different than a regression model without interaction effects [67]. And a multi-layered neural network can be seen as a generalization of regression to account for non-linearities [67]. On the other hand, often methods from statistics and from machine learning tend to give profoundly different results even when they have many commonalities. For example, classical regression fitted with Principal Component Analysis to reduce dimensionality, gives different results that regression fitted with regularization [68]. The latter can also provide means to reduce features, not just dimensions. Or, in another example that is very close to our approach in the present work, regression used with propensity scoring to calculate effects of causal intervention can give very different results from regression used with covariates selected by do-calculus from a causal graph model of the same data [39]. They both fit a regression model but with very different strategies. Eventually all methods, from statistics, applied mathematics, machine learning, etc., are converging into a single new field – that of Data Science.

Our methodology exemplifies this integration through the capacity to find a Markov Boundary using a causal graph method (or a direct Markov Boundary induction method). In this way, we can fit a conventional regression statistical model that typically outperforms the same regression fitted with a weaker feature selector and also has stronger causal interpretation. Our results showed this concretely.

## Conclusion

The results in this first application of ML algorithms to childhood PTSD are consistent with theoretical expectations about the operating characteristics of such methods and provided data to support that PTSD can be predicted to a significant degree from information available shortly after a trauma. Moreover, the integration of causal discovery algorithms within a ML framework can suggest promising strategies for preventative intervention. And several of the causal variables revealed in the present study offer new promise for prevention. The study limitations discussed leave open the possibility for even better models and markers in future studies.

Our data support the notion that Machine Learning techniques of both predictive and causal flavors have significant potential for enhancing the methodological toolkit in the field of childhood PTSD and, more broadly, psychiatry.

## Additional files

Additional file 1: Description of Variables Measured in Study. (DOCX 39 kb)

Additional file 2: Dataset. (TXT 50 kb)

Additional file 3: Technical Notes. (DOCX 121 kb)

## Abbreviations

AUC: Area under the curve (in this study under the ROC curve)
GLM: Generalized Linear Model
MB: Markov Boundary (and less technically strictly: Markov Blanket)
ML: Machine learning
PC: Parents and Children (set)
PTSD RI: Posttraumatic Stress Disorder Reaction Index
RF: Random Forest
RNNCV: Repeated Nested N-fold Cross Validation
ROC: Receiver Operating Characteristic (curve)
SNP: Single-Nucleotide Polymorphism
SVM: Support Vector Machine

## References

[1] Costello EJ, Erkanli A, Fairbank JA, Angold A (2002). The prevalence of potentially traumatic events in childhood and adolescence. J Trauma Stress.

[2] Copeland WE, Keeler G, Angold A, Costello EJ (2007). Traumatic events and posttraumatic stress in childhood. Arch Gen Psychiatry.

[3] Alisic E, Zalta AK, Van Wesel F, Larsen SE, Hafstad GS, Hassanpour K, Smid GE (2004). Rates of post-traumatic stress disorder in trauma-exposed children and adolescents: meta-analysis. Br J Psychiatry.

[4] Giaconia R, Reinherz H, Silverman A, Bilge P, Frost A, Cohen E. Traumas and posttraumatic stress disorder in a community population of older adolescents.1995;34:1369–1380. 10.1097/00004583-199510000-00023 7592275

[5] Santiago PN, Ursano RJ, Gray CL, Pynoos RS, Spiegel D, Lewis-Fernandez R, Friedman MJ, Fullerton CS (2013). A systematic review of PTSD prevalence and trajectories in DSM-5 defined trauma exposed populations: intentional and non-intentional traumatic events. PLoS One.

[6] American Psychiatric Association. Diagnostic and Statistical Manual of Mental Disorders (5^th^ ed.). Arlington, VA: American Psychiatric Publishing; 2013.

[7] Fairbank JA, Putnam FW, Harris WW. The prevalence and impact of child traumatic stress. Handbook of PTSD: Science and Practice. 2007:229–51.

[8] Springer KW, Sheridan J, Kuo D, Carnes M (2007). Long-term physical and mental health consequences of childhood physical abuse: results from a large population-based sample of men and women. Child Abuse Negl.

[9] De Bellis MD, Keshavan MS, Clark DB, Casey BJ, Giedd JN, Boring AM, Ryan ND (1999). Developmental traumatology part II: brain development. Biol Psychiatry.

[10] Trickey D, Siddaway AP, Meiser-Stedman R, Serpell L, Field AP (2012). A meta-analysis of risk factors for post-traumatic stress disorder in children and adolescents. Clin Psychol Rev.

[11] Cox CM, Kenardy JA, Hendrikz JK (2008). A meta-analysis of risk factors that predict psychopathology following accidental trauma. J Spec Pediatr Nurs.

[12] Ray B, Henaff M, Ma S, Efstathiadis E, Peskin ER, Picone M, Poli T, Aliferis CF, Statnikov A (2014). Information content and analysis methods for multi-modal high-throughput biomedical data. Sci Rep.

[13] Statnikov A, Wang L, Aliferis CF (2008). A comprehensive comparison of random forests and support vector machines for microarray-based cancer classification. BMC Bioinformatics.

[14] Dupuy A, Simon RM (2007). Critical review of published microarray studies for cancer outcome and guidelines on statistical analysis and reporting. J Natl Cancer Inst.

[15] Aliferis CF, Statnikov A, Tsamardinos I, Schildcrout JS, Shepherd BE, Harrell FE (2009). Factors influencing the statistical power of complex data analysis protocols for molecular signature development from microarray data. PLoS One.

[16] Narendra V, Lytkin NI, Aliferis CF, Statnikov A (2011). A comprehensive assessment of methods for de-novo reverse-engineering of genome-scale regulatory networks. Genomics.

[17] Statnikov A, Lytkin NI, McVoy L, Weitkamp JH, Aliferis CF (2010). Using gene expression profiles from peripheral blood to identify asymptomatic responses to acute respiratory viral infections. BMC Res Notes.

[18] Statnikov A, Alekseyenko AV, Li Z, Henaff M, Perez-Perez GI, Blaser MJ, Aliferis CF (2013). Microbiomic signatures of psoriasis: feasibility and methodology comparison. Sci Rep.

[19] MAQC Consortium (2010). The MicroArray quality control (MAQC)-II study of common practices for the development and validation of microarray-based predictive models. Nat Biotechnol.

[20] Cabrerizo M, Ayala M, Goryawala M, Jayakar P, Adjouadi M (2012). A new parametric feature descriptor for the classification of epileptic and control EEG records in pediatric population. Int J Neural Syst.

[21] Viangteeravat T (2013). Potential identification of pediatric asthma patients within pediatric research database using low rank matrix decomposition. J Clin Bioinform.

[22] Kukar M, Kononenko I, Grošelj C, Kralj K, Fettich J (1999). Analysing and improving the diagnosis of ischaemic heart disease with machine learning. Artif Intell Med.

[23] Chong SL, Liu N, Barbier S, Ong ME (2015). Predictive modeling in pediatric traumatic brain injury using machine learning. BMC Med Res Methodol.

[24] Mueller A, Candrian G, Kropotov JD, Ponomarev VA, Baschera GM. Classification of ADHD patients on the basis of independent ERP components using a machine learning system. Nonlinear Biomed Phys. 4(Suppl 1). 2010; S1. 10.1186/1753-4631-4-S1-S1 PMC2880795 20522259

[25] Wall DP, Kosmicki J, Deluca TF, Harstad E, Fusaro VA (2012). Use of machine learning to shorten observation-based screening and diagnosis of autism. Transl Psychiatry.

[26] Bedi G, Carrillo F, Cecchi GA, Slezak DF, Sigman M, Mota NB, et al. Automated analysis of free speech predicts psychosis onset in high-risk youths. NPJ Schizophrenia. 2015;1 10.1038/npjschz.2015.30 PMC4849456 27336038

[27] Karstoft KI, Galatzer-Levy IR, Statnikov A, Li Z, Shalev AY (2015). Bridging a translational gap: using machine learning to improve the prediction of PTSD. BMC Psychiatry.

[28] Guyon I, Elisseeff A. An introduction to variable and feature selection. J Mach Learn Res. 2003:1157–82.

[29] Tsamardinos I, Aliferis CF. Towards principled feature selection: relevancy, filters and wrappers. AISTATS. 2003;

[30] Fennessey J (1968). The general linear model: a new perspective on some familiar topics. Am J Sociology.

[31] McNeil KA, Newman I (1996). Kelly FJ.

[32] Friston KJ, Holmes AP, Worsley KJ, Poline JP, Frith CD, Frackowiak RS (1994). Statistical parametric maps in functional imaging: a general linear approach. Hum Brain Mapp.

[33] Mardia KV (1971). The effect of nonnormality on some multivariate tests and robustness to nonnormality in the linear model. Biometrika.

[34] Mohri M, Rostamizadeh A (2012). Talwalkar A.

[35] Michalski RS, Tecuci G. Machine learning: a Multistrategy approach: Morgan Kaufmann; 1994.

[36] Bishop CM (2006). Pattern recognition and machine learning.

[37] Guyon I, Aliferis C, Elisseeff A. Causal feature selection. Computational methods of feature selection. 2007:63–82.

[38] Glymour CN, Cooper GF (1999). Computation, causation, and discovery.

[39] Pearl J (2000). Causality: models, reasoning, and inference.

[40] Granger CWJ (1980). Testing for causality: a personal viewpoint. J Econ Dyn Control.

[41] Aliferis CF, Statnikov A, Tsamardinos I, Mani S, Koutsoukos X (2010). Local causal and Markov blanket induction for causal discovery and feature selection for classification part I:algorithms and empirical evaluation. J Mach Learn Res.

[42] Aliferis CF, Statnikov A, Tsamardinos I, Mani S, Koutsoukos XD (2010). Local causal and Markov blanket induction for causal discovery and feature selection for classification part ii: analysis and extensions. J Mach Learn Res.

[43] Galatzer-Levy IR, Karstoft KI, Statnikov A, Shalev AY (2014). Quantitative forecasting of PTSD from early trauma responses: a machine learning application. J Psychiatr Res.

[44] Steinberg AM, Brymer MJ, Decker KB, Pynoos RS (2004). The University of California at Los Angeles post-traumatic stress disorder reaction index. Curr Psychiatry Rep.

[45] Statnikov A, Aliferis CF, Hardin DP, Guyon I. A gentle introduction to support vector machines in biomedicine: volume 1: theory and methods: World Scientific Publishing Co Inc; 2011.

[46] Bradley A (1997). The use of the area under the ROC curve in the evaluation of machine learning algorithms. Pattern Recogn.

[47] Boser BE, Guyon IM, Vapnik VN (1992). A training algorithm for optimal margin classifiers.

[48] Breiman L (2001). Random Forest. Mach Learn.

[49] Friedman N, Linial M, Nachman I, Pe'er D (2000). Using Bayesian networks to analyze expression data. J Comput Biol.

[50] Bartista G, Monard M (2005). An analysis of four missing data treatment methods for supervised learning. AIJ. 2003;17(5-6):519-33.Saxe GN, Stoddard F, Hall E, Chawla N, Lopez C, Sheridan R, Yehuda R. Pathways to PTSD, part I: children with burns. Am J Psychiatry.

[51] Saxe GN, Stoddard F, Hall E, Chawla N, Lopez C, Sheridan R, Yehuda R (2005). Pathways to PTSD, part I: children with burns. Am J Psychiatry.

[52] De Young AC, Hendrikz J, Kenardy JA, Cobham VE, Kimble RM (2014). Prospective evaluation of parent distress following pediatric burns and identification of risk factors for young child and parent posttraumatic stress disorder. J Child Adolesc Psychopharmacol.

[53] Binder EB, Bradley RG, Liu W, Epstein MP, Deveau TC, Mercer KB, Schwartz AC (2008). Association of FKBP5 polymorphisms and childhood abuse with risk of posttraumatic stress disorder symptoms in adults. JAMA.

[54] Boscarino JA, Erlich PM, Hoffman SN, Zhang X (2012). Higher FKBP5, COMT, CHRNA5, and CRHR1 allele burdens are associated with PTSD and interact with trauma exposure: implications for neuropsychiatric research and treatment. Neuropsychiatr Dis Treat.

[55] Amstadter AB, Nugent NR, Yang BZ, Miller A, Siburian R, Moorjani P, Haddad S, Basu A, Fagerness J, Saxe G, Smoller JW (2011). Corticotrophin-releasing hormone type 1 receptor gene (CRHR1) variants predict posttraumatic stress disorder onset and course in pediatric injury patients. Dis Markers.

[56] Koenan KC, Saxe G, Purcell S, Smoller JW, Bartholomew D, Miller A, Baldwin C (2005). Polymorphisms in FKBP5 are associated with peritraumatic dissociation in medically injured children. Mol Psychiatry.

[57] Charney DS (2004). Psychobiological mechanisms of resilience and vulnerability: implications for successful adaptation to extreme stress. Am J Psychiatry.

[58] Saxe GN, Ellis BH, Brown AD (2016). Survival circuits: how traumatic stress is about survival-in-the-moment (pg 23–45). In trauma systems therapy for children and teens.

[59] Liberty K, Tarren-Sweeney M, Macfarlane S, Basu A, Reid J. Behavior problems and post-traumatic stress symptoms in children beginning School: a comparison of pre- and post-earthquake groups. PLOS Currents Disasters. 2016; doi:10.1371/currents.dis.2821c82fbc27d0c2aa9e00cff532b402. 10.1371/currents.dis.2821c82fbc27d0c2aa9e00cff532b402 PMC5419821 28503358

[60] Goenjian AK, Walling D, Steinberg AM, Roussos A, Goenjian HA, Pynoos RS (2009). Depression and PTSD symptoms among bereaved adolescents 6½ years after the 1988 spitak earthquake. J Affect Disord.

[61] Daniels LA, Magarey A, Battistutta D, Nicholson JM, Farrell A, Davidson G, Cleghorn G (2009). The NOURISH randomised control trial: positive feeding practices and food preferences in early childhood-a primary prevention program for childhood obesity. BMC Public Health.

[62] Gartner LM, Morton J, Lawrence RA, Naylor AJ, O'Hare D, Schanler RJ, Eidelman AI (2005). Breastfeeding and the use of human milk. Pediatrics.

[63] Holder MD, Coleman B, Wallace JM (2010). Spirituality, religiousness, and happiness in children aged 8–12 years. J Happiness Stud.

[64] Houskamp BM, Fisher LA, Stuber ML (2004). Spirituality in children and adolescents: research findings and implications for clinicians and researchers. Child Adolesc Psychiatr Clin N Am.

[65] Reich W (2000). Diagnostic interview for children and adolescents (DICA). J Am Acad Child Adolesc Psychiatry.

[66] aan het Rot M, Collins KA, Murrough JW, Perez AM, Reich DL, Charney DS, Mathew SJ (2010). Safety and efficacy of repeated-dose intravenous ketamine for treatment-resistant depression. Biol Psychiatry.

[67] Duda RO, Hart PE, Stork DG (1973). Pattern classification (Vol. 2).

[68] Friedman J, Hastie T, Tibshirani R (2001). The elements of statistical learning (Vol. 1).

