# Supplementary material for: Machine learning methods to predict child posttraumatic stress: a proof of concept study
Source: BMC Psychiatry. 2017 Jul 10;17:223. doi: 10.1186/s12888-017-1384-1 (PMC5502325; doi:10.1186/s12888-017-1384-1)
Supplement: Supplementary file 1 — Description of Variables Measured in Study. (DOCX 39 kb) [file 12888_2017_1384_MOESM1_ESM.docx]

**Additional File 1: Description of Variables Measured in Study**

This supplement provides details about the variables measured in this study. As detailed in the method section, this study uses an existing data set to predict whether an acutely injured child would have a high level of PTSD symptoms 3 months after hospital discharge. To conduct this predictive analysis, 105 variables that were collected while the child was in the hospital - within days after the injury - were considered. These 105 variables are considered as *features* in the Machine Learning (ML) analysis. The PTSD variable is considered the *target* and is defined as a score of 38 or greater on the UCLA PTSD Reaction Index. All 105 features were included in the ML predictive classification study to test Hypothesis 1. The causal feature selection method was used to test Hypothesis 2 and results indicated that 71 causal features were selected. These 71 causal features are shown in Figure 3 of the main article. In Figure 3, the 11 features that appeared in at least 20 bootstrapped samples are noted and these 11 most stable features are reviewed in the discussion. In the table below, we indicate the 71 features/variables included in the causal predictive model with an asterisk notation *****. The 11 most stable features are notes with ******. Some variables measured at the time of acute hospitalization assessed information about the child and family from periods prior to the injury. These are described with the prefix: ‘Prior’.

The following measures were used in this assessment:

- Diagnostic Interview for Children and Adolescents (DICA) ^1^: a structured diagnostic interview based on the DSM-IV for children ages 6-17.
- Child Behavior Checklist (CBCL)^2^: a 140-item parent-report questionnaire to evaluate behavioral and emotional problems and competencies in children up to age 18.
- Injury Severity Score (ISS)^3^: a brief, well-validated index of injury which is highly correlated to mortality, days spent in hospital, and major surgery.
- Child Dissociative Checklist (CDC) ^4^: a 20-item parent/observer-report measure to evaluate dissociative symptoms in children ages 5-12.
- Child Depression Inventory (CDI) ^5^: a 28-item self-report interval scale to assess symptoms of depression in children ages 7-17 years.
- Coddington Life Events Scale (LES) ^6^: a 36-item self-report measure to assess how specific life events have affected a child’s growth and development.
- Multidimensional Anxiety Scale for Children (MASC) ^7^: a 39-item self-report measure addressing pediatric anxiety symptoms.
- FACES Pain Rating Scale ^8^: a visual analogue interval scale for children ages 3 and up to indicate level of pain.
- Colored Analogue Pain Scale (CAS)^9^: a visual analogue interval scale for children ages 5 and older to indicate level of pain.
- Peritraumatic Dissociation Scale ^10^: an unpublished 18-item interval scale to measure acute peritraumatic dissociation.
- Piers Harris Self-Concept Scale 2 (PH2) ^11^: a 60-item self-report interval scale to assess self-concept in children ages 7-18 years.
- UCLA PTSD Reaction Index (PTSDRI) ^12^: a structured interview for trauma exposure and DSM-IV PTSD symptoms in children ages 7-17.
- Child Stress Reaction Checklist (CSRC) ^13^: a 30-item parent-report measure to assess child traumatic stress.
- Family Strains Scale ^14^: a 15-item interval scale to assess stressful changes within a family.
- Brief Symptom Inventory (BSI) ^15^: a 53-item self-report measure of a broad range of psychiatric symptoms. Administered to the child’s parents to measure their own level of symptoms.
- PTSD Checklist (PCL)^16^:  is a 17-item self-report measure of the 17 DSM-IV symptoms of PTSD. Administered to the child’s parents to measure their own level of posttraumatic symptoms.
- Stanford Acute Stress Reaction Questionnaire^17^: a 31-item scale to measure acute levels of anxiety and dissociative reactions to traumatic events, following DSM-IV criteria for Acute Stress Disorder. Administered to the child’s parents to measure their own level of acute stress.

Additionally, candidate gene analyses and 8am cortisol and MHPG analyses were conducted. The description of measurement of each variable is described in the table below:

**Table 1: Variables and their measurement**

| **Variable name** | **Variable description** | **Measurement** |
| --- | --- | --- |
|  | **FEATURES** | |
| alcohol | Pregnancy ETOH* | How frequently mother drank anything with alcohol during pregnancy as recorded on the 49-item Pregnancy section of the Diagnostic Interview for Children and Adolescents (**DICA**) |
| mostdrin | Pregnancy Drink Number | Mother’s maximum number of alcoholic drinks at one time during pregnancy as recorded on the 49-item Pregnancy section of the **DICA** |
| smokenum | Pregnancy Smoke | Number of cigarettes mother smoked during pregnancy as recorded on the 49-item Pregnancy section of the **DICA** |
| emotion | Pregnancy Emotional Problems | Emotional problems (depression, anxiety) during pregnancy for which mother sought treatment or counseling as recorded on the 49-item Pregnancy section of the **DICA** |
| pregtot | Pregnancy Total Problems* | Mother’s total number of problems during pregnancy as recorded on the 49-item Pregnancy section of the **DICA** |
| gender | Sex* | Child’s sex (male/female) |
| birthwht | Birth Weight* | Child’s birth weight in pounds and ounces as recorded on the 8-item Birth section of the **DICA** |
| prematur | Prematurity | Birth of child was premature as recorded on the 8-item Birth section of the **DICA** |
| hapbaby | Happy Baby* | How happy child was as a baby/whether child gave few problems as recorded on the 12-item Infancy section of the **DICA** |
| diffbaby | Difficult Baby | Child was difficult during 1^st^ year as recorded on the 12-item Infancy section of the **DICA** |
| breast | Breast** Feeding | Whether child was breastfed as recorded on the 12-item Infancy section of the **DICA** |
| crawl | Crawl | Age in months at which child first crawled as recorded on the 12-item Early Development section of the **DICA** |
| sit_up | Sit Up | Age in months at which child first sat up as a baby as recorded on the 12-item Early Development section of the **DICA** |
| walk | Walk | Age in months at which child first walked as recorded on the 12-item Early Development section of the **DICA** |
| crying | Crying | How much child cried (was never satisfied) as a baby as recorded on the 12-item Infancy section of the **DICA** |
| sleep | Sleep | Parent had trouble getting child to sleep at night as recorded on the 12-item Sleep Disorders section of the **DICA** |
| panic | Panic | Child was troubled by panic states in the middle of the night as recorded on the 12-item Sleep Disorders section of the **DICA** |
| words | Words | Age in months at which child first used understandable words as recorded on the 12-item Early Development section of the **DICA** |
| sentence | Sentences | Age in months at which child first used complete sentences as recorded on the 12-item Early Development section of the **DICA** |
| kinder | Kindergarten* | Child attended kindergarten as recorded on the 16-item Year-One-Through-Six section of the **DICA** |
| totprobs | Dev Probs* | Total number of child developmental problems as recorded on the 16-item Year-One-Through-Six section of the **DICA** |
| heldback | Held Back* | Child has been held back in school as recorded on the 31-item Demographics section of the **DICA** |
| iep | IEP* | Child has had or has Individual Education Plan as recorded on the 31-item Demographics section of the **DICA** |
| playspor | Sports* | Child plays a sport as recorded on the 22-item demographics section of the **DICA** |
| worship | Religious Service** | Child attends church, synagogue, or other place of worship as recorded on the 22-item demographics section of the **DICA** |
| sick | Sick | Child ever sick/ injured or had to go to hospital or stay home a long time as recorded on the 31-item Demographics section of the **DICA** |
| ptsdpass | Prior PTSD** | Child’s past PTSD symptoms, as recorded on the 24-item PTSD section of the **DICA** |
| talk2som | Help Seeking** | Child talked to someone (counselor, doctor, rabbi, priest, etc.) about troubles as recorded on the 22-item demographics section of the **DICA** |
| ses | SES | Socioeconomic status of child’s family as recorded on the Child Behavior Checklist (**CBCL**) |
| peoplehm | People in Home* | Number of people that live in child’s home as recorded on the 31-item Demographics section of the **DICA** |
| age | Age at Trauma | Age of child in years at time of trauma as recorded on child’s medical record |
| head | Head Injury* | Child suffered head injury as recorded on child’s medical record |
| inj_vio | Violent Injury* | Child suffered violent injury (stabbing, assault, gunshot wound) or non-violent injury (all others) as recorded on child’s medical record |
| iss | Injury Severity* | Child’s Injury Severity Score as recorded by child’s admitting surgeon on child’s medical record |
| days | Hosp Stay | Length of stay in hospital in days following injury as recorded on child’s medical record |
| newasddx | ASD Diagnosis | Whether child was diagnosed with Acute Stress Disorder (ASD) from the ASD Module of the DICA [20] |
| cbclact | Prior Activity Competence* | Child’s score on the Activities Competence Scale of the **CBCL** about the child prior to injury (parent rating) |
| cbclsch | Prior School Competence* | Child’s score on the School Competence Scale of the **CBCL** about the child prior to injury (parent rating) |
| cbclsoc | Prior Social Competence* | Child’s score on the Social Competence Scale of the **CBCL** about the child prior to injury (parent rating) |
| cbclext | Prior Externalizing** | Child’s Externalizing score on the **CBCL** about the child prior to injury (parent rating) |
| cbclint | Prior Internalizing* | Child’s Internalizing score on the **CBCL** about the child prior to injury (parent rating) |
| cbclptsd | Prior PTSD (parent rating) | Child’s PTSD Scale score on the CBCL about the child prior to injury (parent rating) |
| cdc | Prior Dissociation* | Child’s score on the Child Dissociative Checklist (**CDC**) about the child prior to injury (parent rating) |
| cditot | Acute Depression | Child’s total score on the Child Depression Inventory (**CDI**) about the child in hospital (child rating) |
| les_death | Prior Stress/Death** | Child’s sum of death questions on the Coddington Life Events Interval Scale (**LES**) (parent rating) |
| les_family | Prior Stress/ Family | Child’s sum of family stress questions on the **LES** |
| les_school | Prior Stress/School* | Child’s sum of school questions on the **LES** |
| lestot | Prior Stress Total* | Child’s total score on the **LES** |
| mascha | Acute Harm Avoidance* | Child’s score on the Harm Avoidance Scale of the Multidimensional Anxiety Interval Scale for Children (**MASC**) about the child in hospital |
| mascsa | Acute Social Anxiety* | Child’s score on the Social Anxiety Scale of the **MASC** about the child in hospital |
| mascsep | Acute Separation Anxiety* | Child’s score on the Separation Anxiety Scale of the **MASC** about the child in hospital |
| masctot | Acute Anxiety Total* | Child’s total score on the **MASC** about the child in hospital |
| fps | Acute Pain (FPS)* | Child’s score on the Facial Pain Scale (**FPS**) |
| painva | Acute Pain (VAS)** | Child’s Pain Visual Analogue Scale (VAS) score collected by interactive measure (Colored Analogue Pain Interval Scale) during acute hospitalization following injury |
| perit | Acute Dissociation* | Total score on the Peritraumatic Dissociation Interval Scale about the child in hospital |
| ph2pa | Body Image* | Child’s score on the Physical Appearance and Attributes Interval Scale of the Piers-Harris Children’s Self-Concept Interval Scale, 2^nd^ Ed. (**PH2**) during acute hospitalization following injury |
| ph2sat | Happiness and Satisfaction | Child’s score on the Happiness and Satisfaction Interval Scale of the **PH2** during acute hospitalization following injury |
| ph2tot | Self-Esteem* | Child’s total score on the **PH2** during acute hospitalization following injury |
| csrcptot | Child Acute Stress (Parent)* | Child’s score on the Child Stress Disorders Checklist (CSDC), rated by parent during acute hospitalization following injury |
| csrcntot | Child Acute Stress (Nurse)* | Child’s score on the Child Stress Disorders Checklist (CSDC), rated by the child’s nurse during acute hospitalization following injury |
| strainsc | Prior Family Stress | Family score on the **Family Strains Scale** about the child prior to injury (parent rating) |
| bsidept | Parent Acute Depression | Parent’s own depression score on the Brief Symptom Inventory (**BSI**) during child’s acute hospitalization following injury |
| bsipsdit | Parent Acute Emotional Symptoms | Parent’s own positive symptoms score on the **BSI** during acute hospitalization following child’s injury |
| bsigsit | Parent Acute Global Severity | Parent’s own Global Severity Index T-score on the **BSI** during acute hospitalization following child’s injury |
| stantot | Parent Acute Stress | Parents total score on the Stanford Acute Stress Questionnaire. |
| ADRB153 | ADRB153 | ADRB153: SNP rs1801253 on the adrenoceptor beta (ADRB) gene analyzed via buccal DNA samples obtained via mouthwash, isolated using Gentra DNA isolation kit, and typed using real-time PCR technology |
| ADRB178 | ADRB178 | ADRB178: SNP rs2183378 on the adrenoceptor beta (ADRB) gene analyzed via buccal DNA samples obtained via mouthwash, isolated using Gentra DNA isolation kit, and typed using real-time PCR technology |
| AVPR1a15 | AVPR1a15 | AVPR1a15: SNP rs1042615 on the arginine vasopressin receptor 1a (AVPR1a) gene analyzed via buccal DNA samples obtained via mouthwash, isolated using Gentra DNA isolation kit, and typed using real-time PCR technology |
| AVPR1a20 | AVPR1a20 | AVPR1a20: SNP rs11174820 on the arginine vasopressin receptor 1a (AVPR1a) gene analyzed via buccal DNA samples obtained via mouthwash, isolated using Gentra DNA isolation kit, and typed using real-time PCR technology |
| AVPR1a39 | AVPR1a39 | AVPR1a39: SNP rs10784339 on the arginine vasopressin receptor 1a (AVPR1a) gene analyzed via buccal DNA samples obtained via mouthwash, isolated using Gentra DNA isolation kit, and typed using real-time PCR technology |
| AVPR1a46 | AVPR1a46 | AVPR1a46: SNP rs11836346 on the arginine vasopressin receptor 1a (AVPR1a) gene analyzed via buccal DNA samples obtained via mouthwash, isolated using Gentra DNA isolation kit, and typed using real-time PCR technology |
| AVPR1a50 | AVPR1a50 | AVPR1a50: SNP rs2738250 on the arginine vasopressin receptor 1a (AVPR1a) gene analyzed via buccal DNA samples obtained via mouthwash, isolated using Gentra DNA isolation kit, and typed using real-time PCR technology |
| AVPR1a54 | AVPR1a54 | SNP rs2228154 on the arginine vasopressin receptor 1a (AVPR1a) gene analyzed via buccal DNA samples obtained via mouthwash, isolated using Gentra DNA isolation kit, and typed using real-time PCR technology |
| COMT33 | COMT33 | COMT33: SNP rs4633 on the catechol-O-methyltransferase (COMT) gene analyzed via buccal DNA samples obtained via mouthwash, isolated using Gentra DNA isolation kit, and typed using real-time PCR technology |
| COMT69 | COMT69 | COMT69: SNP rs6269 on the catechol-O-methyltransferase (COMT) gene analyzed via buccal DNA samples obtained via mouthwash, isolated using Gentra DNA isolation kit, and typed using real-time PCR technology |
| CRHR104 | CRHR104 | CRHR104: SNP rs17763104 on the corticotropin releasing hormone receptor 1 (CRHR1) gene analyzed via buccal DNA samples obtained via mouthwash, isolated using Gentra DNA isolation kit, and typed using real-time PCR technology |
| CRHR112 | CRHR112 | CRHR112: SNP rs12944712 on the corticotropin releasing hormone receptor 1 (CRHR1) gene analyzed via buccal DNA samples obtained via mouthwash, isolated using Gentra DNA isolation kit, and typed using real-time PCR technology |
| CRHR114 | CRHR114 | CRHR114: SNP rs17690314 on the corticotropin releasing hormone receptor 1 (CRHR1) gene analyzed via buccal DNA samples obtained via mouthwash, isolated using Gentra DNA isolation kit, and typed using real-time PCR technology |
| CRHR142 | CRHR142 | CRHR142: SNP rs242942 on the corticotropin releasing hormone receptor 1 (CRHR1) gene analyzed via buccal DNA samples obtained via mouthwash, isolated using Gentra DNA isolation kit, and typed using real-time PCR technology |
| CRHR144 | CRHR144 | CRHR144: SNP rs4458044 on the corticotropin releasing hormone receptor 1 (CRHR1) gene analyzed via buccal DNA samples obtained via mouthwash, isolated using Gentra DNA isolation kit, and typed using real-time PCR technology |
| CRHR158 | CRHR158 | CRHR158: SNP rs17763658 on the corticotropin releasing hormone receptor 1 (CRHR1) gene analyzed via buccal DNA samples obtained via mouthwash, isolated using Gentra DNA isolation kit, and typed using real-time PCR technology |
| CRHR161 | CRHR161 | CRHR161: SNP rs4074461 on the corticotropin releasing hormone receptor 1 (CRHR1) gene analyzed via buccal DNA samples obtained via mouthwash, isolated using Gentra DNA isolation kit, and typed using real-time PCR technology |
| CRHR181 | CRHR181 | CRHR181: SNP rs12936181 on the corticotropin releasing hormone receptor 1 (CRHR1) gene analyzed via buccal DNA samples obtained via mouthwash, isolated using Gentra DNA isolation kit, and typed using real-time PCR technology |
| CRHR192 | CRHR192 | CRHR192: SNP rs11657992 on the corticotropin releasing hormone receptor 1 (CRHR1) gene analyzed via buccal DNA samples obtained via mouthwash, isolated using Gentra DNA isolation kit, and typed using real-time PCR technology |
| FKBP502 | FKBP502 | FKBP502: SNP rs4713902 on the FK506 binding protein 5 (FKBP5) gene analyzed via buccal DNA samples obtained via mouthwash, isolated using Gentra DNA isolation kit, and typed using real-time PCR technology |
| FKBP524 | FKBP524 | FKBP524:SNP rs9380524 on the FK506 binding protein 5 (FKBP5) gene analyzed via buccal DNA samples obtained via mouthwash, isolated using Gentra DNA isolation kit, and typed using real-time PCR technology |
| FKBP533 | FKBP533 | FKBP533: SNP rs6926133 on the FK506 binding protein 5 (FKBP5) gene analyzed via buccal DNA samples obtained via mouthwash, isolated using Gentra DNA isolation kit, and typed using real-time PCR technology |
| FKBP534 | FKBP534 | FKBP534: SNP rs10498734 on the FK506 binding protein 5 (FKBP5) gene analyzed via buccal DNA samples obtained via mouthwash, isolated using Gentra DNA isolation kit, and typed using real-time PCR technology |
| FKBP542 | FKBP542 | FKBP542: SNP rs17614642 on the FK506 binding protein 5 (FKBP5) gene analyzed via buccal DNA samples obtained via mouthwash, isolated using Gentra DNA isolation kit, and typed using real-time PCR technology |
| FKBP547 | FKBP547 | FKBP547: SNP rs3777747 on the FK506 binding protein 5 (FKBP5) gene analyzed via buccal DNA samples obtained via mouthwash, isolated using Gentra DNA isolation kit, and typed using real-time PCR technology |
| FKBP558 | FKBP558 | FKBP558: SNP rs9296158 on the FK506 binding protein 5 (FKBP5) gene analyzed via buccal DNA samples obtained via mouthwash, isolated using Gentra DNA isolation kit, and typed using real-time PCR technology |
| FKBP563 | FKBP563 | FKBP563: SNP rs10947563 on the FK506 binding protein 5 (FKBP5) gene analyzed via buccal DNA samples obtained via mouthwash, isolated using Gentra DNA isolation kit, and typed using real-time PCR technology |
| FKBP573 | FKBP573 | FKBP573: SNP rs3800373 on the FK506 binding protein 5 (FKBP5) gene analyzed via buccal DNA samples obtained via mouthwash, isolated using Gentra DNA isolation kit, and typed using real-time PCR technology |
| OPRM171 | OPRM171 | OPRM171: SNP rs1799971 on the opioid receptor, mu 1 (OPMR1) gene analyzed via buccal DNA samples obtained via mouthwash, isolated using Gentra DNA isolation kit, and typed using real-time PCR technology |
| dbp | Acute Diastolic BP* | Average diastolic blood pressure over hospital stay as recorded by child’s nurse on child’s medical record |
| sbp | Acute Systolic BP* | Average systolic blood pressure over hospital stay as recorded by child’s nurse on child’s medical record |
| pulse | Acute Pulse* | Pulse during entire length of hospital stay as recorded on child’s medical record |
| cortisolbase | 8am Cortisol* | Blood sample collected via Qaigen Kits and shipped to Children’s Hospital Boston (CHB) Molecular Genetics Core Facility for cortisol analysis |
| benzo | Acute Benzo* | Benzodiazepine use (mg/kg/total) during total length of hospital stay as recorded on child’s medical record |
| ketamine | Acute Ketamine** | Ketamine use (mg/kg/total) during total length of hospital stay as recorded on child’s medical record |
| morphine | Acute Morphine | Morphine use (mg/kg/total) during total length of hospital stay as recorded on child’s medical record |
| bsbandva | Acute Heart Variance -Baseline | Heart beat-to-beat variability at baseline stress test. Measured by Minimeter physiologic monitor. Stress procedure involved exposing child to 3 sequential 1 minute video clips. The first video clip was a neutral pleasant clip. The second exposed the child to a clip reminiscent of their injury. The third repeated the neutral clip. Heart Var-Base was the measure of heart beat-to-beat variability during the neutral video clip.^18-19^ |
| stbandva | Acute Heart Variance-Stress | Heart Var-Stress was the measure of heart beat-to-beat variability during the 1 minute video clip reminiscent of the child’s injury, in the Stress Procedure defined above. ^18-19^ |
| rcbandva | Acute Heart Variance-Recovery | Heart Var-Recovery was the measure of heart beat-to-beat variability during the presentation of the 1minute neural video clip that followed the 1minute stress video clip, in the Stress Procedure defined above. ^18-19^ |
| meanmhpg | Mean MHPG Values* | Mean MHPG levels collected via Oragene kit and shipped to CHB Molecular Genetics Core Facility |
|  | **TARGET** | |
| ptsdrito | **Child PTSD** | Child’s score on the UCLA PTSD Reaction Index administered to the child 3 months after injury |

**References**

1. Reich W. Diagnostic interview for children and adolescents (DICA). J Am Acad Child Adolesc Psychiatry. 2000; 39(1): 59-66.
2. Achenbach TM. Manual for the Child Behavior Checklist 4-18 and 1991 Profile. Burlington, VT: University Associates in Psychiatry. 1991.
3. Baker SP, O’Neill B, Haddon W, Long WB. The injury severity score: a method for describing patients with multiple injuries and evaluating emergency care. J Trauma. 1974; 14(3): 189-96.
4. Putnam FW, Peterson G. Further validation of the Child Dissociative Checklist. Dissociation: Progress in the Dissociative Disorders. 1994; 7(4): 204-11.
5. Kovacs M. Children’s Depression Inventory (CDI) Technical Manual. New York, NY: Multi-Health Systems, Inc. 2001.
6. Coddington RD. The significance of life events as etiological factors in the diseases of children: II. A study of a normal population. J Psychosomatic Res. 1972; 16: 205-13.
7. March JS, Parker JD, Sullivan K, Stallings P, Conners CK. The Multidimensional Anxiety Scale for Children (MASC): factor structure, reliability, and validity. J Am Acad Child Adolesc Psychiatry. 1997; 36(4): 554-65.
8. Bieri D, Reeve R, Champion G, Addicoat L, Ziegler J. The Faces Pain Scale for the self-assessment of the severity of pain experienced by children: development, initial validation and preliminary investigation for ration scale properties. Pain. 1990; 41: 139-50.
9. Bulloch B, Garcia-Filion P, Notricia D, Bryson M, McConahay T. Reliability of the Color Analog Scale: Repeatability of Scores in Traumatic and Nontraumatic Injuries. Acad Emerg Med. 2009; 16(5): 465-9.
10. Saxe, G. Peritraumatic Dissociation Interval Scale. Unpublished.
11. Piers EV. Piers-Harris 2: Piers Harris Children’s Self-Concept Scale, 2^nd^ Ed. Los Angeles, CA: WPS Publishers. 2002.
12. Pynoos RS, Frederick C, Nader K, Arroyo W, Steinberg A, Eth S, et al. Life Threat and Posttraumatic Stress in School-Age Children. Arch Gen Psychiatry. 1987; 44(12): 1057-63.
13. Saxe G, Chawla N, Stoddard F, Kassam-Adams N, Courtney D, Cunningham K, et al. Child Stress Disorders Checklist: A Measure of ASD and PTSD in Children. J Amer Acad Child Adolesc Psychiatry. 2003; 42(8): 972-8.
14. Moos RHM. Family Environment Scale Manual: Development, applications, research, 3^rd^ Ed. Palo Alto, CA: Consulting Psychologists Press Inc. 1994.
15. Derogatis LR. BSI Brief Symptom Inventory: Administration, Scoring, and Procedure Manual, 4^th^ Ed. Minneapolis, MN: National Computer Systems Pearson, Inc. 1993.
16. Blanchard EB, Jones-Alexander J, Buckley TC, Forneris CA. Psychometric properties of the PTSD checklist (PCL). Behav Res Ther. 1996; 34: 669-73.
17. Cardeña E, Koopman C, Classen C, Waelde LC, Spiegel D. Psychometric properties of the Stanford Acute Stress Reaction Questionnaire (SASRQ): a valid and reliable measure of acute stress. J Trauma Stress. 2000; 13(4): 19-34.
18. Porges SW. Vagal tone: A physiologic marker of stress vulnerability*.* Pediatrics. 1992; 90(3): 498-504.
19. Porges SW. Orienting in a defensive world: Mammalian modifications of our evolutionary heritage. A polyvagal theory*.* Psychophysiology. 1995; 32: 301-18.
20. Miller A, Enlow MB, Reich W, Saxe G. A diagnostic interview for acute stress disorder for children and adolescents. J Trauma Stress. 2009; 22(6): 549-56.
