# Supplementary material for: Machine learning methods to predict child posttraumatic stress: a proof of concept study
Source: BMC Psychiatry. 2017 Jul 10;17:223. doi: 10.1186/s12888-017-1384-1 (PMC5502325; doi:10.1186/s12888-017-1384-1)
Supplement: Supplementary file 3 — Technical Notes. (DOCX 121 kb) [file 12888_2017_1384_MOESM3_ESM.docx]

**Additional File 3: Technical Notes**

**Technical note on interpretation of HITON-PC output and causal validity of identified variables**

Recall that the Markov Boundary of the response variable is the smallest set of variables that contain all predictive information about the response variable contained in the data. In the majority of distributions it has been shown that the Markov Boundary has also a causal interpretation since it contains the direct causes, the direct effects, and the direct causes of the direct effects (i.e., so-called “spouses”) of the response variable. HITON-PC in general is not designed to find the full Markov Boundary of the response variable but only its direct causes and direct effects. An extension of HITON-PC, called HITON-MB finds also spouse variables and thus returns the complete Markov Boundary. Because the response variable (PTSD) in our study is a terminal variable, it only has direct causes but no direct effects or spouses. Thus the Markov Boundary will be the set of direct causes. An indirect but very informative way to test whether the produced set by HITON-PC is indeed the set of direct cases is therefore to test that this set contains all information of the variables in the dataset (because it is also the Markov Boundary as explained). Indeed, the best signal attained by using all variables is 0.79 AUC, whereas the best signal with HITON-PC features is 0.74 AUC, This implies that any direct causes of PTSD not identified by HITON-PC have very marginal significance and that the identified set is indeed a very good approximation of the MB and therefore it is a very close approximation of the set of direct causes of PTSD (in support of Hypothesis 2 of the present study).

**Technical note on interpretation of SVM model weights**

In the results section we presented the weights of features in the best model. While the magnitude of weights (w) for the features in the SVM model are very informative as to the effect of the features to the model’s output, unfortunately they can also be misleading in the sense that they do not denote information overlap. In other words, two equally informative features in the model A, and B may have similar information and identical weights, however we could eliminate one of the two without loss of predictivity. Stated in a slightly more technical language, the SVM reject (i.e. minimize w of) irrelevant features) but retain *weakly relevant features* and spread the weights among them [1]. The weakly relevant features are extremely misleading with regards to causality however [2]. When the SVM model is fitted with a feature selector that retains only strongly relevant features and rejects weakly relevant ones (as HITON does) then the interpretation of weights is only with regards to local causal or Markov Boundary features.

1. Hardin, D., Tsamardinos, I. and Aliferis, C.F., 2004, July. A theoretical characterization of linear SVM-based feature selection. In *Proceedings of the twenty-first international conference on Machine learning* (p. 48). ACM.
2. Statnikov, A., Hardin, D. and Aliferis, C., 2006. Using SVM weight-based methods to identify causally relevant and non-causally relevant variables. *sign*, *1*(4).


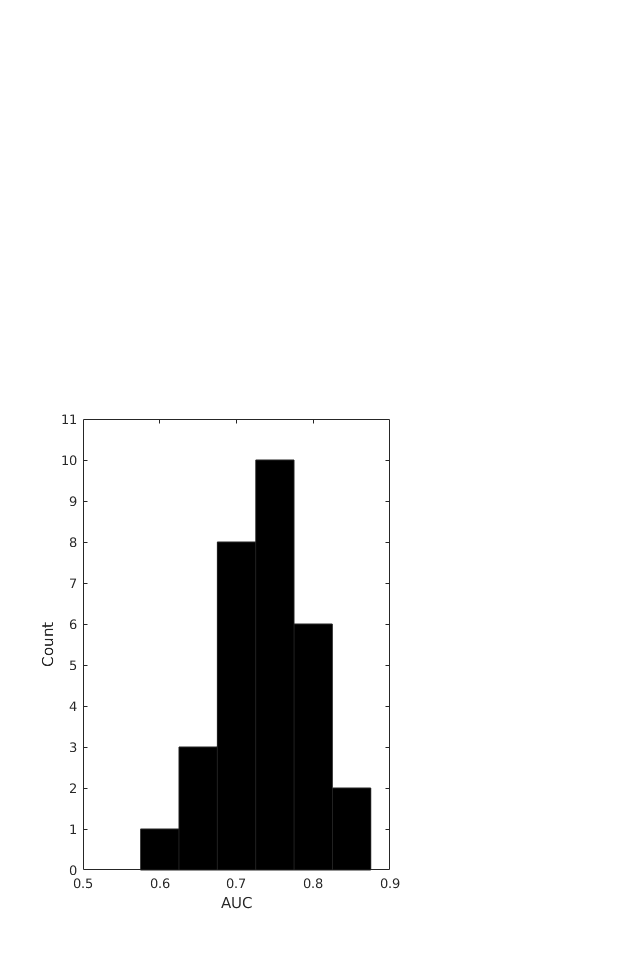


**Figure 5**. **Distribution of mean AUC (averaged over five fold cross validation) obtained from 30 repeats.** Results shown for Lasso with HITON-PC features. Other models result in similar distributions.
